# The environmental adaptation of acidophilic archaea: promotion of horizontal gene transfer by genomic islands

**DOI:** 10.1186/s12864-025-11875-5

**Published:** 2025-07-28

**Authors:** Jingxuan Qiu, Huiling Tao, Hongyu Li, Xinyi Liu, Rui Liu, Muhammed Naveed Nawaz, Xingjie Wang, Liyuan Ma

**Affiliations:** 1 School of Environmental Studies, China University of Geosciences, Wuhan, 430074 China; 2 School of Water Science, University of Waterloo, Waterloo, N2L3G1 Canada; 3 School of Engineering, Cardiff University, Cardiff, CF243AA UK

**Keywords:** Genomic island, Horizontal gene transfer, Archaea, Acid mine drainage, Environmental adaptation

## Abstract

Acid mine drainage (AMD) is an extremely acidic leachate highly contaminated with metal ions, yet it harbors a significantly high abundance of archaea. Genomic islands (GIs), as one of the productions of horizontal gene transfer (HGT), play an important role in the environmental adaptation and evolutionary processes of archaea. However, the distribution, structure, and function of GI within the genomes of archaea remain poorly understood. In this study, through the bioinformatic analysis of archaea in AMD, including *Ferroplasma acidiphilum* ZJ isolated from laboratory and 25 acidophilic archaea collected from NCBI database, 176 GIs were predicted and annotated. Furthermore, we analyzed their structural features and provided insights into the role of HGT in environmental adaptation. The size and distribution of GIs in the genomes were found to be random. In the majority of GIs, the GC content was lower than the average GC content of the strain genome, suggesting that GIs were typically looped out of the genomes with poor stability and transferred into those with higher stability. tRNAs with classical stem-loop secondary structures have been found at the ends of several GIs, suggesting that GIs frequently integrate near tRNAs. In contrast to functional genes directly involved in cellular life processes, GIs were more likely to carry genes related to genetic information and metabolism. Several GIs were identified to carry genes involved in iron oxidation, mercury reduction, and various toxin-antitoxin systems, which enhance the adaptability of the strains to highly acidic environments.

**Supplementary Information:**

The online version contains supplementary material available at 10.1186/s12864-025-11875-5.

## Introduction

Previous phylogenetic studies have revealed that life on Earth consists of three primary domains, i.e., bacteria, archaea, and eukarya [1, 2]. Nevertheless, with the discovery of the Asgard superphylum (including Loki-. Thor-, Odin- and Heimdallarchaeota) in the past few years [3, 4], James Lake’s two-domain theory which includes only bacteria and archaea has gained more and more support [5]. Archaea, the third form of life, provides valuable insights into the early stages of evolution. The cell morphology and structure of archaea are similar to that of bacteria, but the genetic information transfer systems such as genome replication, transcription, and translation are closer to those of eukaryotes [6]. These organisms thrive in a variety of habitats, especially extreme environments, which show tremendous diversity in genetics and metabolism and drive the cycling of elements on Earth. Archaea not only colonize diverse environments ranging from the human gut) to agricultural soils [7, 8], but uniquely retain dominance in extreme habitats. Acid mine drainage (AMD) is an exceptionally acidic and highly contaminated leachate generated from underground operations of closed or abandoned mine sites and the accumulation of tailings or mullocks [9]. It has been demonstrated that at pH < 2, the relative abundance of archaea in AMD systems increases to 25% of all prokaryotes belonging to this domain [10]. However, our understanding of archaeal biology remains far less than that of bacteria, primarily due to the challenges in isolating and culturing these microorganisms. Some researchers have engineered and operated a methane-fed, continuous-flow bioreactor system for over 2,000 days to enrich archaea from deep-marine methane-seep sediments [11].

The adaptive mechanisms underlying survival in extreme acidic environments remain a persistent focus in extremophile research, particularly regarding evolutionary pathways. A substantial proportion of bacterial and archaeal genetic diversity originates from sequence acquisitions from other environmental microbes [12].

Horizontal gene transfer (HGT), also referred as lateral gene transfer, is defined as the movement of genetic material between distinct species or across substantial phylogenetic distances by non-vertical inheritance mechanisms [13]. HGT is neutral and, in principle, any gene may be transferred via HGT. Such transfers can confer advantageous traits related to ecological niche adaptation or pathogenicity but may also introduce numerous non-beneficial genes, imposing a metabolic burden on the recipient microorganisms [14–16]. Mobile genetic elements (MGEs) act as key vectors that facilitate the frequency and genomic scope of HGT. Through specialized mechanisms such as conjugation, transduction, and transposition, MGEs mediate the transfer of adaptive traits (e.g., antibiotic resistance) while simultaneously propagating themselves across phylogenetically distant taxa [17]. MGEs offer several benefits to microorganisms. However, the maintenance of MGEs imposes a considerable fitness cost on hosts [18, 19].

In 1990, researchers identified clusters of virulence genes present in the genomes of certain *Escherichia coli* strains. These clusters, recognized as a type of MGEs, were named genomic islands (GIs) [20]. It has been reported that GIs initially enter the new strain through HGT before being integrated into the host chromosome via site-specific recombination. Following integration, GIs may evolve through processes such as gene rearrangement, and the loss or acquisition of new mobile genes [21]. GIs have special sequences and structural features that set them apart from the rest of genomes: sporadic distribution, instability, ability to excise spontaneously, sequence composition bias, atypical codon usage, large size, proximity to tRNA genes, and flanking direct repeats (DRs) [22]. However, not all genomic regions that meet the above characteristics can be called GIs, but the more criteria a sequence meets, the more likely it is a product of HGT. Moreover, GIs carry genes that can promote the adaptation of their host to its specific ecological niches, such as pathogenesis, symbiosis, novel metabolic pathways, and resistance to antibiotics, prophages, or heavy metal cations [23].

To better understand the environmental adaptability of archaea in AMD, the GIs of an archaeal *Ferroplasma acidiphilum* ZJ isolated from AMD of Zijin Mine and 25 other acidophilic archaeal strains collected from NCBI were predicted. Furthermore, we analyzed the GIs’ structure to investigate the correlation between structure and mobility. Additionally, the function of genes in the GIs was also annotated to investigate their positive impacts on the host’s adaptation to the environment.

## Materials and methods

### Data collection

The isolation process involved inoculating 10.0 mL aliquots of liquid samples into modified 9 K medium (pH 1.6) supplemented with 22.4 g/L FeSO_4_·7H2O and 0.02% (w/v) yeast extract. Cultivation was conducted at 40 °C under aerobic conditions using a rotary shaker at 170 rpm. The base 9 K medium composition consisted of (per liter): (NH_4_)_2_SO_4_ (3.00), K_2_HPO_4_ (0.50), KCl (0.10), Ca(NO_3_)_2_ (0.01), and MgSO_4_·7H_2_O (0.50). The DNA of *F. acidiphilum* ZJ was sequenced with a combination of PacBio RS and Illumina HiSeq platform supported by Shanghai Majorbio Bio-pharm Technology Co., Ltd. (Shanghai, China).

Previous studies have investigated 25 strains of archaea that have been identified in AMD. They belong to 6 genera including *Thermoplasmatales* (6), *Ferroplasma* (6), *Acidiplasma* (5), *Candidatus Parvarchaeota* (3), *Candidatus Mancarchaeum* (1), *Cuniculiplasma* (2), *Thermogymnomonas* (2) (Table S1). For this study, the data, including genomic sequences, genomic size, accession number, isolation source, genomic status, and GC content of 25 strains mentioned above, were downloaded from the NCBI (https://www.ncbi.nlm.nih.gov/). The phylogenetic tree of the above 26 strains of acidophilic archaea was shown in the Figure S1.

### GIs identification

A wide array of methods has been developed to predict and visualize GIs, the majority of which depend on recognizing the structural features and nucleotide composition of GIs. First published in 2009, Island Viewer was the inaugural web server to integrate four of the most accurate and complementary GI prediction tools: IslandPick, IslandPath-DIMOB, SIGI-HMM, and Islander [24]. IslandPath-DIMOB utilizes nucleotide bias and the presence of mobility genes to identify GIs, while SIGI-HMM employs a Hidden Markov Model approach to address codon usage bias. IslandPick, in contrast, adopts a comparative genomic methodology to pinpoint GIs. Researchers compared IslandViewer4 with other GI prediction tools and found that it has attracted much interest because of its high accuracy and specificity [25]. Thus, this study used IslandViewer4 to predict the potential GIs. The reference genome for GI prediction was *Ferroplasma acidarmanus* fer1. The virulence factor homologs in GIs were identified in close relatives of genomes with curated data based on a reciprocal best blast hit (RBBH) approach with very stringent cutoff values: e-value cutoff of 1e-10, > 90% sequence similarity, and > 80% coverage. Newly added predictions from Islander based on the frequent use of tRNA and tmRNA genes as integration sites [26].

### Structure of GIs

The sequences of GIs were obtained from IslandViewer4. The GC Content Calculator, a web-based tool, was utilized to calculate the exact percentage of GC content in predicted GIs. The tRNA genes near the predicted GIs were identified with tRNAscanSE2.0 [27]. The archaeal genomes were selected as the sequence source and the search mode was set at default.

RNAfold was used to predict the secondary structure of tRNA. The multiple sequence alignment (MSA) of flanked tRNA was analyzed by MUSCLE [28]. The fold algorithms were minimum free energy (MFE) and partition function. The basic option was avoiding isolated base pairs. The dangling end option was dangling energies on both sides of a helix in any case. The energy parameters were RNA parameters (Turner model, 2004).

### Function of GIs

Genes within the predicted GIs were annotated using EggNOG5.0, which classifies genes into three taxonomic levels; the bacterial subset of COGs, archaeal arCOGs and eukaryotic KOGs. The input sequence type was protein and other parameters were default. The COG system categorizes the data into 26 functional groups. Several of these categories describe functions primarily associated with eukaryotic cells. The recently added V (Defense mechanisms) and X (Mobilome) categories provide a more detailed description of the dynamics of bacterial and archaeal genomes. Functional categories are assigned by the cellular roles of the respective COGs [29].

## Results and discussion

### Distribution and quantity of GIs in AMD archaea genomes

**Fig. 1:**
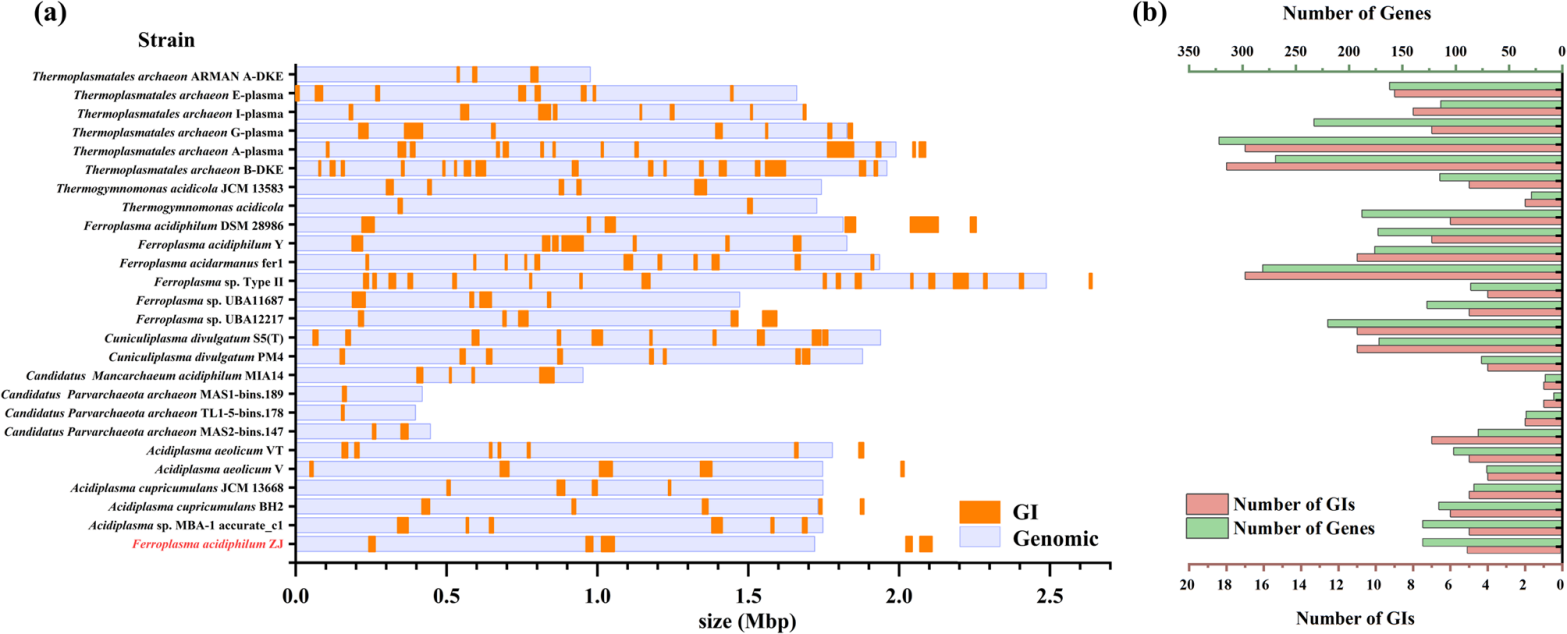
**a** Distribution map of GIs in genome. **b** The number of GIs in each strain and the number of genes contained in GIs of each strain

One hundred thirty-one genes total amongst 5 GIs were predicted in *F. acidiphilum* ZJ. A total of 171 GIs were identified across the genomes of other 25 acidophilic archaea. Among these 26 archaea, two strains, namely *Mancarchaeum acidiphilum* MIA14 and *Candidatus Parvarchaeota archaeon* TL1-5-bins.178, were identified by IslandPath-DIMOB, and the rest were identified using SIGI-HMM (Table S2). The length of GIs ranged from 4,215 bp to 91,819 bp, with 71.3% falling between 8 kb and 40 kb. The positions of GIs were random in most strains’ genomes (Fig. 1a). The significant differences in the distribution and number of GIs in the archaeal genomes may be due to the large differences in the size and sequencing quality of the 26 archaeal genomes. It can be noted that IslandViewer4 defaulted to splicing multiple contigs with N in the process of GI prediction so that the GIs of several strains extended beyond the end of their genomes. The lowest had only one GI, while the highest had 18 GIs among 25 genomes. The total number of genes contained in the GIs varied among genomes too. The genome of TL1-5-bins.178, with only a single GI, contained the fewest genes within its GIs, numbering only 8. In contrast, a total of 322 genes were annotated in *Thermoplasmatales archaeon* A-plasm’s GIs (Fig. 1b).

**Fig. 2:**
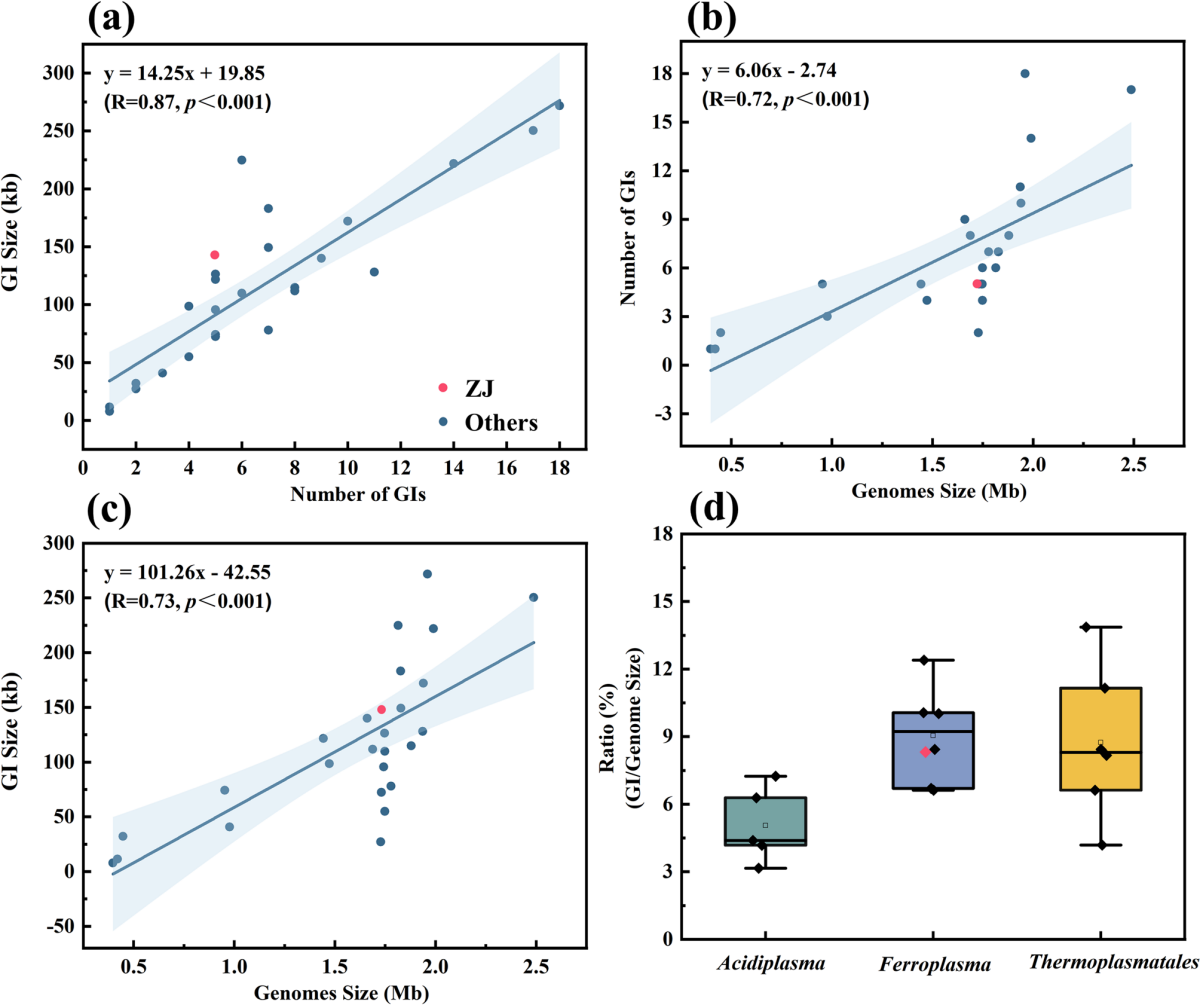
Patterns of GIs in AMD archaea genomes. **a** Linear regression analysis between number of GIs per archaea genome with GI size (in kb). **b** Relationship between genome size and number of GIs. **c** Relationship between archaea genome and GI size. And **d** Box and whiskers graphic of the GI ratio for the AMD archaea

The analysis between the number of GIs and the size of all GIs in every single genome revealed a Pearson correlation coefficient of 0.87 (*p*-value < 0.001), indicating a strong positive linear correlation (Fig. 2a). Moreover, linear regression analysis also confirmed a positive linear relationship between the genome size and the number of all GIs or size of GIs (Fig. 2b and c). However, compared to archaea with the same number of GIs, ZJ had the highest total size of GIs (Fig. 2a). *F. acidiphilum* ZJ had a lower number of GIs than archaea with similar genome sizes (Fig. 2b). This is consistent with the “genome expansion hypothesis” proposed by McDaniel et al. that HGT increases both total genome length and GIs accumulation [30]. Among the three major genera, *Acidiplasma* exhibited a lower ratio of the total GIs sequence size and host genome size (5.05%), while the genera *Ferroplasma* and *Thermoplasmatales* had about 9.04% and 8.74%, respectively (Fig. 2d). Archaea of the genus *Ferroplasma* exhibited a higher efficiency of GIs integration, suggesting a specific role of genomic plasticity in heavy metal stress adaptation. This discrepancy may be attributed to the inferior genome assembly of *Acidiplasma* (Table S1). When genome assemblies are fragmented, larger genomic islands may be cut into multiple short contigs, resulting in a prediction algorithm that fails to recognize the complete boundaries, leading to a low predicted GI number. Previous research has also found that the number of GIs varied in different strains of the same species. For example, *Aeromonas hydrophila* ATCC 7966 only contained found that 13 GIs while there were 33 GIs in *Aeromonas hydrophila* NJ-35 [31]. These variations may result from the spontaneous cleavage of GIs, and some GIs were even lost after being cultured in the laboratory [32].

### The secondary structure and multiple sequence alignment of tRNAs flanking GIs

Transfer RNAs (tRNAs) are crucial molecular adaptors that mediate the translation of genetic code. These molecules undergo a variety of post-transcriptional modifications, which enhance their chemical reactivity while simultaneously influencing their structure, stability, and functionality. The tRNA is usually composed of 70–90 nucleotides, and its 3’ end serves as the site for amino acid attachment. As described above, GIs scattered across the archaeal chromosomes. Several studies have indicated that they were often integrated at the 3’ end of tRNA and formed direct repeat sequences (DRs) [23, 33, 34]. The sequence of tRNA is highly conserved, resulting in a low mutation rate during HGT. The secondary structure of tRNA consists of a cloverleaf, and this more stable structure also ensures the low mutability of tRNA to a certain extent [35]. There were two main reasons why tRNA can become the integrase selection site: tRNA was more reliable than other coding genes, and the other was the small size of tRNA facilitates the host genome’s recovery of target genes upon integration [36].

**Fig. 3:**
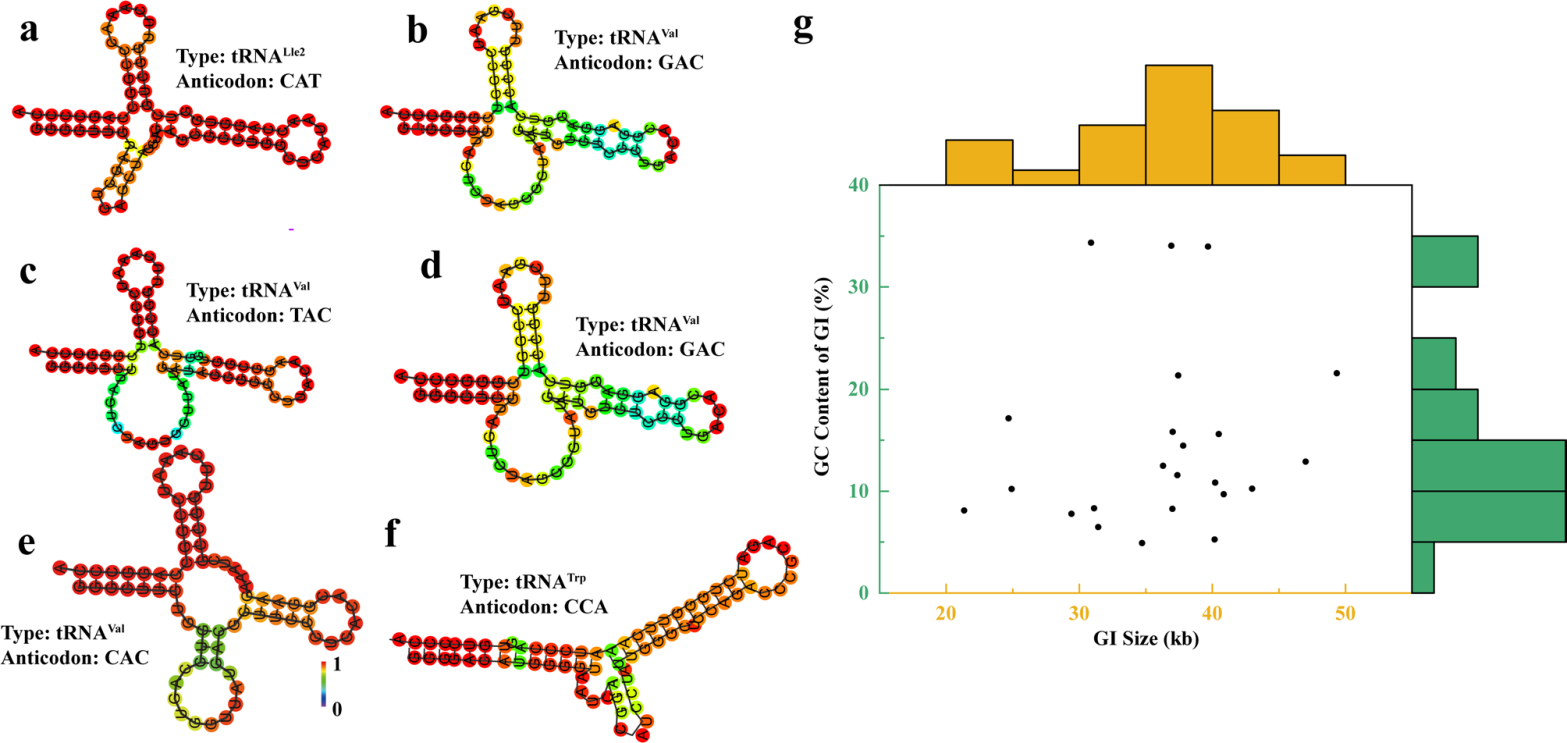
**a**-**f** Secondary structure of flanked tRNAs. **g** Marginal histogram of GI size with flanked tRNA and its GC content

tRNA flanking sequences were observed in archaeal GIs, a hallmark of horizontal gene transfer. Notably, 23 of the 171 predicted GIs (13.5%) exhibited this characteristic (Table S3). The proportion of GIs flanked by tRNA relative to the total number of tRNA in these archaea genomes was 7.3%. The size of tRNAs ranged from 70 to 142 bp. 14 genomes with the flanked tRNA correspond to 9 species of 6 genera. The predicted tRNAs exhibited a high degree of primary and secondary structure diversity (Fig. 3a-f, Table S3 and Fig. S2). All predicted tRNA had the typical cloverleaf secondary structure. The anticodons of these tRNAs correspond to 10 different amino acids, including alanine (Ala), arginine (Arg), cystine (Cys), glycine (Gly), leucine (Leu), isoleucine (Ile), methionine (Met), threonine (Thr), tryptophan (Trp) and valine (Val). Among these, tRNA^Leu^ and tRNA^Val^ accounted for the largest proportion of anticodons. A marginal histogram explored the relationship between GI’s size and GC content and flanked tRNA’s existence. The result indicated that GIs with 5–15% GC content and a size of 30–45 kb had a higher probability of carrying flanked tRNA (Fig. 3g). GIs with low GC content may be retained by the host by maintaining gene expression compatibility through the conservation of flanking tRNAs (e.g., promoters or terminators) when integrating into high GC hosts. Previous studies have pointed out that horizontally transferred DNA often has a different GC content from the host, and flanking conserved sequences (e.g., tRNAs) may buffer transcriptional conflicts arising from such differences [37]. 30–45 kb sized GIs are moderate in size, accommodating a larger cluster of functional genes, and also avoid being eliminated by the host due to the metabolic burden of redundant genes [21].

**Fig. 4:**
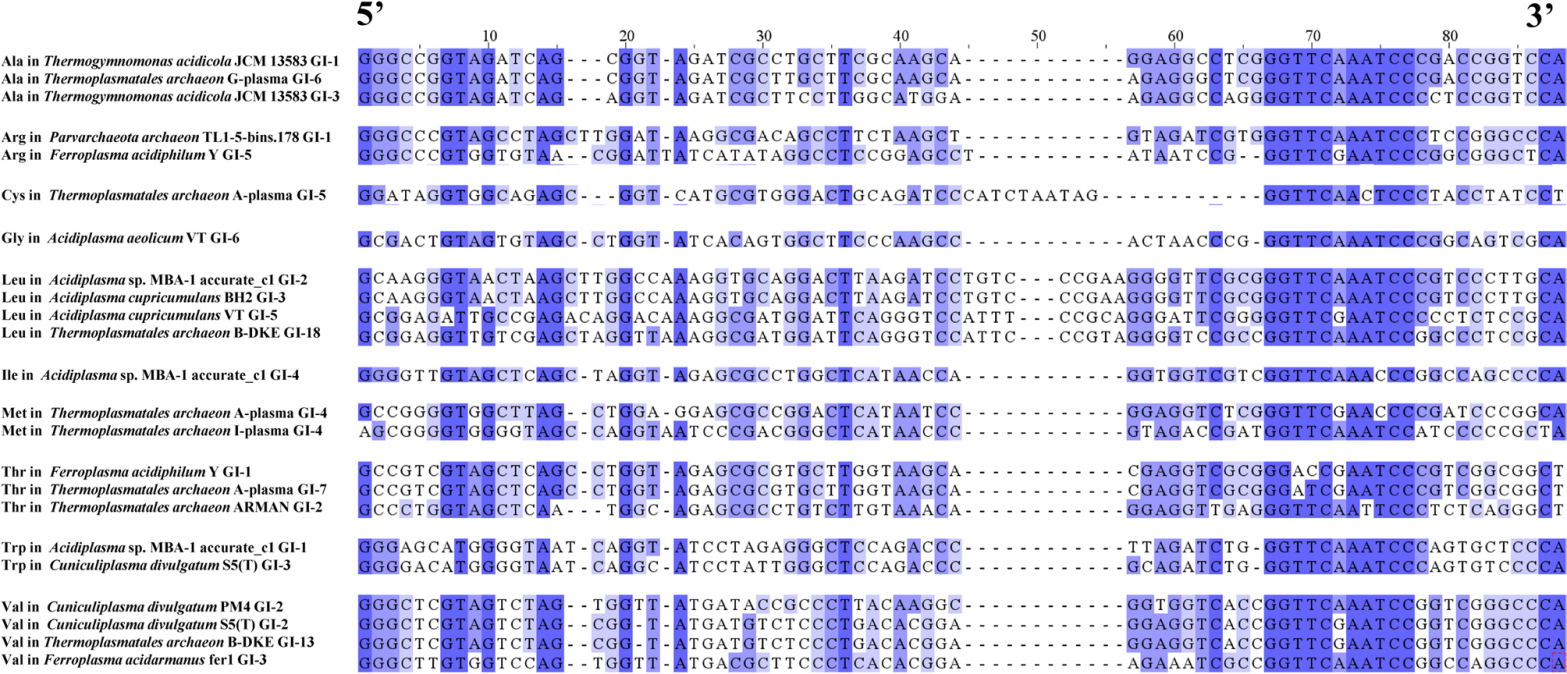
Multiple sequence alignment among the flanked tRNA sequences

Multiple sequence alignment (MSA) provides critical insights into sequence-structure-function relationships within nucleotide or protein sequence families [38]. The findings revealed that the homology of downstream sequences and the similarity of flanking tRNA sequences were greater than those observed upstream (Fig. 4). Additionally, the same tRNA with identical nucleotide sequences and secondary structures may be found in different GIs, such as the same tRNA^Leu^ whose anticodon was TAA at the 2nd GI of *Acidiplasma* sp. MBA-1 accurate_c1 and the 3rd GI of *Acidiplasma cupricumulans* BH2. In addition, the same tRNA^Val^ whose anticodon was GAC at the 2nd GI of *Cuniculiplasma divulgatum* S5(T) and 13th GI of *Thermoplasmatales archaeon* B-DKE.

### GC characterization of GIs and its comparison with the average GC of the host genome

**Fig. 5:**
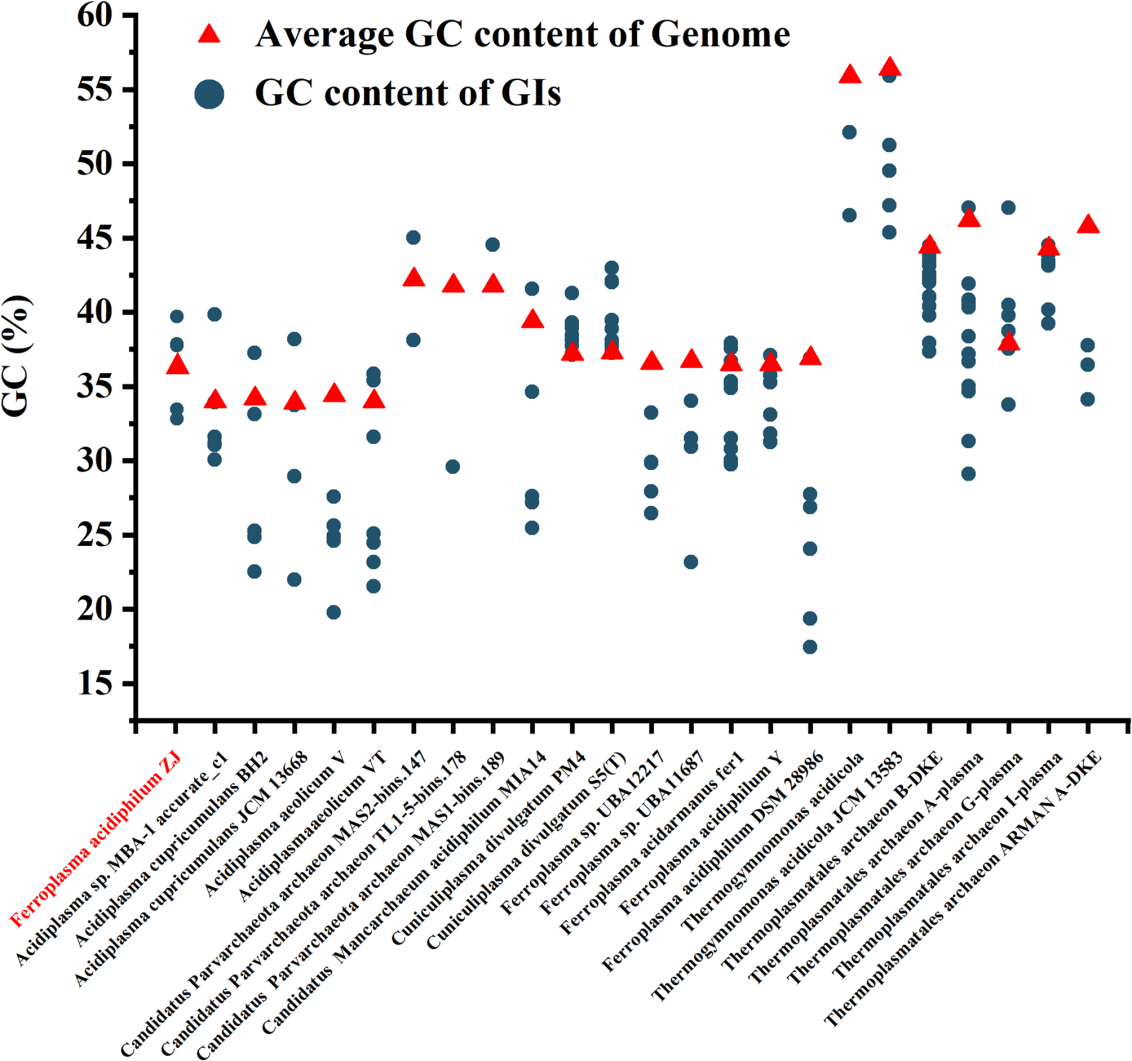
Comparison of average GC content of Genome and GC content of each GI

GC content refers to the proportion or percentage of guanine (G) and cytosine (C) base pairs in a genome. Given its fundamental importance for the maintenance and transfer of genetic information, the diversity of GC content and its evolutionary determinants have been extensively studied over several decades [39]. GC content contributes to the stability of nucleic acids through hydrogen bonding, with increased hydrogen bonds generally leading to greater stability [40]. The average GC content was similar among strains of the same genus (Fig. 5). The GC content of *F. acidiphilum* ZJ genome was 36.9%, and that of the five Gis was 37.1%, 32.3%, 32.9%, 37.0% and 38.9%, respectively. Among the five genera investigated, the average GC content of *Acidiplasma* was the lowest (33.9 − 34.4%), while the average GC content of *Thermogymnomonas* was the highest (55.9 − 57.0%). GC content also varied among strains from 36.5 to 36.9% of *Ferroplasma*, from 37.2 to 37.3% of *Cuniculiplasma*, and from 44.3 to 56.4% of *Thermoplastatales* except for *T. archaeon* G-plasma whose average GC content was lower than other strains.

Compared to the average GC content of the host genome, 75.7% GIs exhibited a lower GC content (Fig. 5). This was consistent with the result of previous researchers [41]. For example, the GC content of PBGI-1 and PBGI-2 was lower than that of *Pseudomonas bharatica* CSV86 [41]. Similarly, Pongchaikul observed that the GC content of AcGI 1 was 61.5%, which was lower than 67.5% of the host genome [42]. GIs, as results of HGT, exhibit reduced stability in archaeal genomes due to their low GC content, which may facilitate integrase-mediated excision for either biased genomic retention or HGT-driven transfer to new hosts. The low GC content of GIs in the host genome results in their lower stability than other regions, which makes them more susceptible to integrase shearing, allowing them to either be biased in the host genome or to be sheared and then removed from the original host and transferred via horizontal gene transfer into the genome of the next host.

### Classification and annotation of genes in GI via COG

**Fig. 6:**
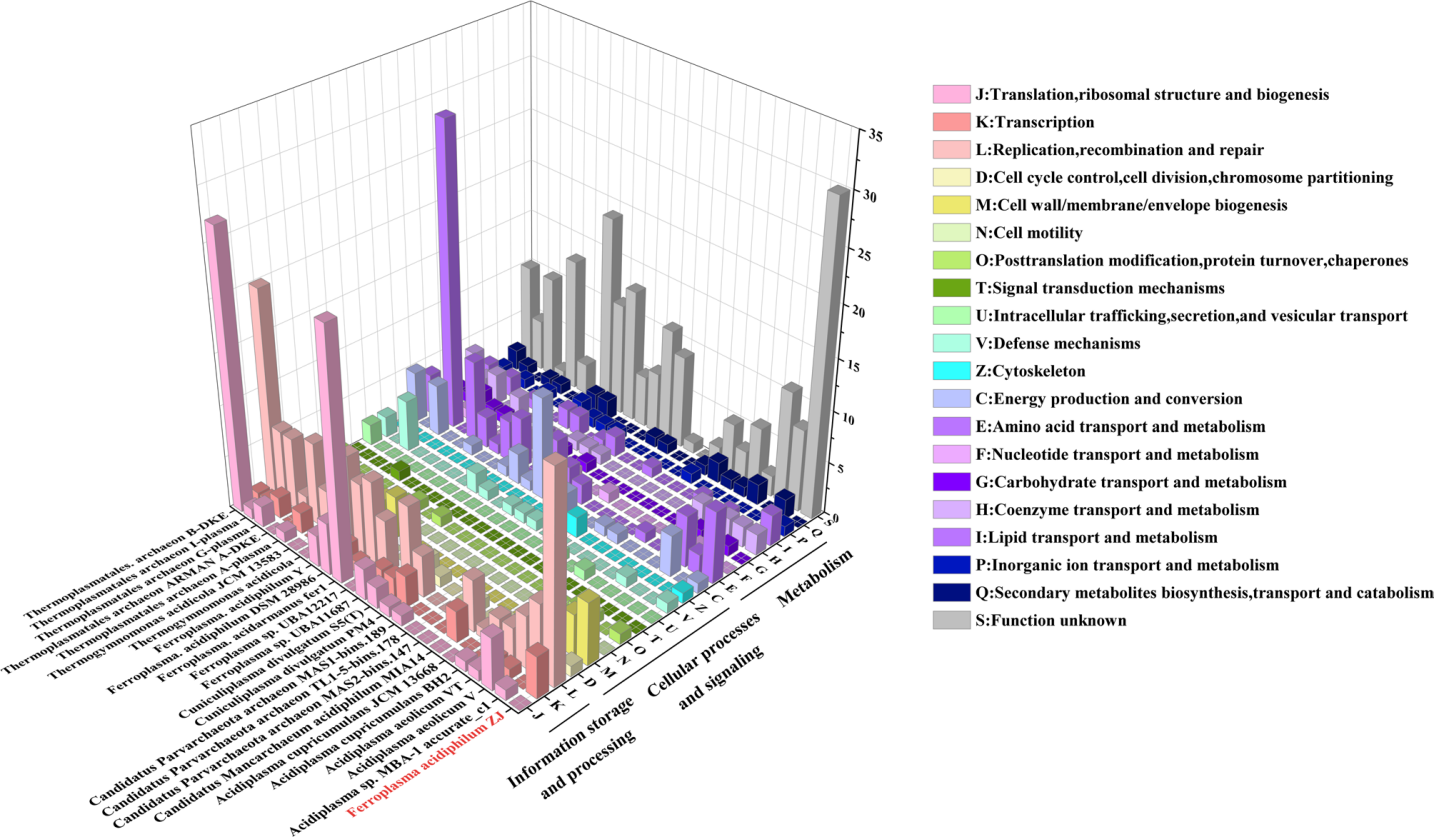
COG classification of genes in GIs. Category J, K and L belong to information storage and processing; category D, M, N, O, T, U, Z, and C belong to cellular processes and signaling; category E, F, G, H, I, P and Q belong to metabolism

37% of all genes on GIs are uncharacterized and part of the mobilome. These unwanted genes cause redundancy in the host genome and increase the metabolic and reproductive burden. A total of 63% of the island genes (673) were annotated using the COG classification. Among these, the majority (11.9%) were associated with replication, recombination, and repair (L), followed by amino acid transport and metabolism (E, 10.1%), carbohydrate transport and metabolism (G, 9.8%), and energy generation and conversion (C, 3.9%) (Fig. 6). Among the GIs of *F. acidiphilum* ZJ, the most functional genes annotated were category L, followed by E and M (cell wall/membrane/envelop biogenesis). Previous studies have indicated that categories C, E, J (translation, ribosomal structure, and biogenesis), along with category L, represent the predominant functional categories in the GIs of acidophilic bacteria [43]. The above four categories belonged to two groups, metabolism and information storage and processing. According to the overall distribution analysis, the majority of the genes were in these two groups, while the genes belonging to the group of cell life processes and signal transduction were quite few. These results indicated that in contrast to genes related to cellular life processes, the GIs were more inclined to carry genes related to genetic information processing and metabolism.

In addition to the above categories, there were two categories without characteristics: R (general function prediction only) and S (function unknown). They reflected the current level of interpretation of protein functions at the proteome level [44]. The results revealed the absence of the R category in the GIs, but the number of S category proteins accounted for 48.6% of the total. This indicated that the nearly half of the genes in the GIs remained uncharacterized, and the structure and function of many proteins were still unclear and needed further exploration and research.

### GIs related to enhance stress resistance

**Fig. 7:**
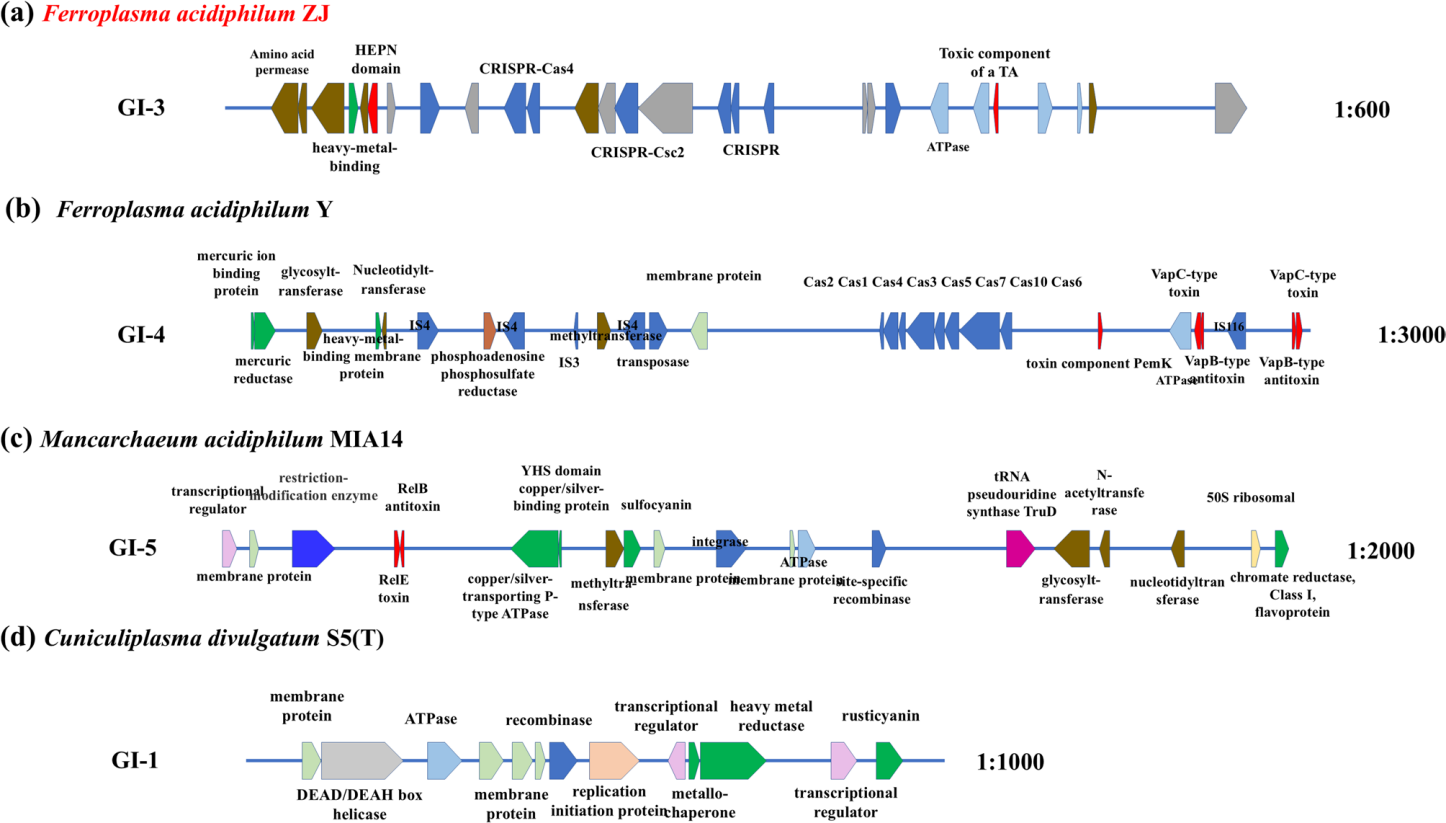
Genetic physical map of GIs related to enhance stress resistance

Since all the material strains in this study are derived from AMD, which is characterized by a high concentration of heavy metals, the GIs with genes related to heavy metal and toxin resistance were studied further. The physical maps of genes in GIs are presented in Fig. 7 and Fig. S3. These GIs contained genes encoding proteins involved in heavy metal metabolism, including Copper/silver-transporting P-type ATPase, YHS domain copper/silver-binding protein, Sulfocyanin, Chromate reductase Class I flavoprotein, Metallochaperone, Rusticyanin, Mercuric ion binding protein, Mercuric reductase, 4Fe-4 S ferredoxin and Heavy-metal-binding membrane protein. CopA, which specifically recognized and confers resistance to Ag(I) and Cu(I) ions, was one of the few known members of the heavy-metal efflux resistance-nodulation-cell division (HME-RND) family [44]. The proteome was composed of CopZ (a cytoplasmic protein that can bind Cu^+^ and deliver it to CopA), CopA, and CusF (can receive Cu^+^ delivered by CopA and transfer it to the CusCFBA efflux system). The CusCFBA efflux system effectively expels Cu^+^ from the cells thus adapting to the high Cu^+^ environment [45]. The introduction of metal-binding proteins, which coordinated and bound functional groups to heavy metal ions through conformational changes, can enhance the metal-binding capacity, metal tolerance, and accumulation ability of the strain [46]. It indicated that CopA was important for heavy metal resistance in *Mancarchaeum acidiphilum* MIA14 inhabiting acidic environments with high concentrations of dissolved metal ions (Fig. 7c) [47]. Furthermore, it was reported that sulfocyanin in the 1th GI of *Cuniculiplasma divulgatum* S5(T) was one of the blue copper-containing proteins which probably played a crucial role in iron oxidation in some archaea and bacteria lineages [48] (Fig. S3).

MazF toxin, MazE antitoxin, RelE toxin, RelB antitoxin, VapC toxin, VapB antitoxin, and the toxin component PemK were proteins involved in toxin resistance. The MazFE, RelEB, and VapCB toxin-antitoxin (TA) were composed of the aforementioned proteins [49] (Fig. 7b, c and Fig. S3). TA systems were initially identified due to their role in plasmid stabilization, as bacterial cells that lose the plasmid following cell division experience either growth arrest or death. Subsequently, TA loci were shown to play a variety of roles, including bacterial cell adaptation to stress, phage resistance, and offering protection against superinfection when encoded within prophage genomes. TA systems commonly are composed of a stable toxin, which inhibits cellular growth, and a labile antitoxin, which counteracts toxicity [49–53]. The MazE antitoxin was directly bound to the MazF toxin and formed a protein-protein complex, resulting in its neutralization. However, MazE was a short-lived protein and would be degraded by proteases of the Clp family or by Lon under stress conditions. After the degradation of MazE, MazF was unleashed from the complex and acted as a sequence-specific endoribonuclease that cleaved RNAs [54–56]. For example, MazF has been reported to cleave the 3’ end of the 16 S ribosomal RNA, thus disrupting its binding to mRNA at the ribosome binding site and inhibiting translation initiation [49]. The RelBE system was recognized as a key component of the bacterial stress response, primarily regulating the global level of translation and function in the quality control of gene expression. The RelE toxin acted as an endoribonuclease, which cleaved ribosome-bound mRNAs between the second and the third base of the A-site codons, resulting in translational inhibition and bacterial cell growth stasis. The direct binding of the RelB antitoxin to RelE neutralized its activity by inducing a conformational change that disrupts toxin structure, thereby alleviating its toxic effects [53, 57, 58]. The VapC was a tRNA endonuclease, and VapB was known to bind to VapC to inhibit its endonuclease activity [59–61]. Under standard conditions, the expression levels of toxin and antitoxin were equivalent because the antitoxin counteracted the harmful effects of the toxin so that the strain could survive normally.

Additionally, a variety of mobile genes, such as integrase, transposase, and site-specific recombinase were identified in within multiple GIs (Fig. S3). These mobile genes were closely related to the mobility of MGEs and, their existence suggests that the GIs may have resulted from spontaneous HGT or the residue of other MGEs. The above three enzymes located within GIs played a crucial role in genomic evolution [62–67]. According to a previous study, site-directed recombination involved tyrosine/serine recombinase or DDE transposase to recognize the flanking repeats, leading to the exchange of DNA segments between integration/excision modules of different GIs [68]. There was also a group of Cas proteins in the 3rd GI of *F. acidiphilum* ZJ and 4th GI of *F. acidiphilum* Y, which, constituted a specific immune defense system known as the CRISPR/Cas system along with CRISPR (Fig. 7a and b). Cas2 and Cas4 genes were predominantly identified, arranged adjacent to CRISPR loci, and encode Cas proteins possessing nuclease, helicase, integrase, and other enzymatic activities. These proteins activities are critical for the recognition and degradation of foreign genetic material [69].

The presence of the above genes brought the host strains stronger metal metabolism and might helped them mitigate the impact of the heavy metal environment. They also enhanced the resistance of the host to the invasion of external substances, such as toxins and viruses. Because of the randomness of HGT, the genes located in the GIs exhibited considerable diversity. Whether they were GIs with genes related to metal element circulation or toxin resistance, the presence of these genes either expanded the host’s metabolic pathway or improved its metabolic efficiency. Ultimately, this process contributed to the enrichment of the genetic diversity of archaea, which highlights the ecological significance of HGT.

## Conclusion

A total of 171 GIs were identified in the genomes of 25 acidophilic archaeal strains. The sizes of GIs ranged from 4,215 bp to 91,819 bp. The size and distribution position of the GIs in the genome did not have an obvious regularity. The GC content of most GIs was lower than the average GC content of the strain whole genome. The tRNA types at the end of GI showed obvious tRNA^Leu^ and tRNA^Val^ bias.

The COG annotation classification resulted in genes in the GIs displayed that among all the annotated genes, the number of replication, recombination, and repair categories was the largest. On the whole, the GIs were more inclined to carry genes related to genetic information and metabolism and less to carry functional genes closely related to cell life processes. Multiple genes related to iron oxidation reactions, mercury ion reduction, and copper metabolism pathways were found in multiple GIs. Additionally, the existence of multiple TA systems and mobile genes improved the adaptability of the strain to the extremely acidic mine environments.

## Supplementary Information

Supplementary Material 1.

Supplementary Material 2.

Supplementary Material 3.

Supplementary Material 4.

Supplementary Material 5.

## Data Availability

(1)Reference strain used for this study listed in TableS1. Genomes data for these strains can be obtained from NCBI. The potential GIs were predicted by IslandViewer4 at https://www.pathogenomics.sfu.ca/islandviewer/. (2) The data, including genomic sequences, genomic size, accession number, isolation source, genomic status, and GC content of 25 strains mentioned above, were downloaded from the NCBI (https://www.ncbi.nlm.nih.gov/). (3) The GC content of GIs was calculated by GC Content Calculator (http://www.endmemo.com/bio/gc.php). (4) The tRNA genes near the predicted GIs were identified with tRNAscanSE2.0 (http://lowelab.ucsc.edu/tRNAscan-SE/). (5) RNAfold (http://rna.tbi.univie.ac.at/cgi-bin/RNAWebSuite/RNAfold.cgi) was used to predict the secondary structure of tRNA. (6) Genes within the predicted GIs were annotated using EggNOG5.0 (http://eggnog5.embl.de/).

## Abbreviations

AMD: Acid mine drainage
GI: Genomic island
HGT: Horizontal gene transfer
DR: Direct repeats
MGEs: Mobile genetic elements
NCBI: National Center for Biotechnology Information
COGs: Clusters of Orthologous Groups of proteins
KOGs: Eukaryotic Orthologous Groups
MSA: Multiple sequence alignment

## References

[1] Woese CR, Kandler O, Wheelis ML. Towards a natural system of organisms: proposal for the domains archaea, bacteria, and eucarya. Proc Natl Acad Sci. 1990;87(12):4576–9. 2112744 10.1073/pnas.87.12.4576 PMC54159

[2] Fox GE, et al. The phylogeny of prokaryotes. Science. 1980;209(4455):457–63. 6771870 10.1126/science.6771870

[3] Banciu HL, et al. Asgard archaea in saline environments. Extremophiles. 2022;26(2):21. 35761090 10.1007/s00792-022-01266-z

[4] Russum S, et al. Comparative population genomic analyses of transporters within the Asgard archaeal superphylum. PLoS ONE. 2021;16(3):e0247806. 33770091 10.1371/journal.pone.0247806 PMC7997004

[5] Lake JA, et al. Eocytes: a new ribosome structure indicates a Kingdom with a close relationship to eukaryotes. Proc Natl Acad Sci. 1984;81(12):3786–90. 6587394 10.1073/pnas.81.12.3786 PMC345305

[6] van Wolferen M, et al. The cell biology of archaea. Nat Microbiol. 2022;7(11):1744–55. 36253512 10.1038/s41564-022-01215-8 PMC7613921

[7] Ruaud A, et al. Syntrophy via interspecies H2 transfer between Christensenella and Methanobrevibacter underlies their global cooccurrence in the human gut. MBio. 2020;11(1):03235–19. 10.1128/mbio. 10.1128/mBio.03235-19 PMC7002349 32019803

[8] Baker BJ, et al. Diversity, ecology and evolution of Archaea. Nat Microbiol. 2020;5(7):887–900. 32367054 10.1038/s41564-020-0715-z

[9] Yang M, et al. Mechanism of acid mine drainage remediation with steel slag: A review. ACS Omega. 2021;6(45):30205–13. 34805655 10.1021/acsomega.1c03504 PMC8600512

[10] Korzhenkov AA, et al. Archaea dominate the microbial community in an ecosystem with low-to-moderate temperature and extreme acidity. Microbiome. 2019;7(1):11. 30691532 10.1186/s40168-019-0623-8 PMC6350386

[11] Imachi H, et al. Isolation of an archaeon at the prokaryote–eukaryote interface. Nature. 2020;577(7791):519–25. 31942073 10.1038/s41586-019-1916-6 PMC7015854

[12] Nelson-Sathi S, et al. Origins of major archaeal clades correspond to gene acquisitions from bacteria. Nature. 2015;517(7532):77–80. 25317564 10.1038/nature13805 PMC4285555

[13] Woods LC, et al. Horizontal gene transfer potentiates adaptation by reducing selective constraints on the spread of genetic variation. Proc Natl Acad Sci. 2020;117(43):26868–75. 33055207 10.1073/pnas.2005331117 PMC7604491

[14] Kurland CG, Canback B, Berg OG. Horizontal gene transfer: a critical view. Proc Natl Acad Sci. 2003;100(17):9658–62. 12902542 10.1073/pnas.1632870100 PMC187805

[15] Yin Z, et al. Horizontal gene transfer clarifies taxonomic confusion and promotes the genetic diversity and pathogenicity of Plesiomonas shigelloides. mSystems. 2020;5(5):10–1128. 10.1128/mSystems.00448-20 PMC7498682 32934114

[16] Li L, et al. Insights into the metabolism and evolution of the genus acidiphilium, a typical acidophile in acid mine drainage. mSystems. 2020;5(6):10–1128. 10.1128/mSystems.00867-20 PMC7677001 33203689

[17] Frost LS, et al. Mobile genetic elements: the agents of open source evolution. Nat Rev Microbiol. 2005;3(9):722–32. 16138100 10.1038/nrmicro1235

[18] Liu Z, et al. Mobile genetic elements mediate the mixotrophic evolution of novel Alicyclobacillus species for acid mine drainage adaptation. Environ Microbiol. 2021;23(7):3896–912. 33913568 10.1111/1462-2920.15543

[19] Callens M, Scornavacca C, Bedhomme S. Evolutionary responses to codon usage of horizontally transferred genes in Pseudomonas aeruginosa: gene retention, amelioration and compensatory evolution. Microb Genomics. 2021;7(6):000587. 10.1099/mgen.0.000587 PMC8461475 34165421

[20] Hacker J, et al. Deletions of chromosomal regions coding for fimbriae and hemolysins occur in vitro and in vivo in various extra intestinal Escherichia coli isolates. Microb Pathog. 1990;8(3):213–25. 1974320 10.1016/0882-4010(90)90048-u

[21] Juhas M, et al. Genomic islands: tools of bacterial horizontal gene transfer and evolution. FEMS Microbiol Rev. 2009;33(2):376–93. 19178566 10.1111/j.1574-6976.2008.00136.x PMC2704930

[22] Feng Y, et al. Using the pan-genomic framework for the discovery of genomic Islands in the Haloarchaeon Halorubrum ezzemoulense. mBio. 2024;15(5):e00408–24. 38619241 10.1128/mbio.00408-24 PMC11078007

[23] Bertelli C, et al. Enabling genomic Island prediction and comparison in multiple genomes to investigate bacterial evolution and outbreaks. Microb Genomics. 2022;8(5):000818. 10.1099/mgen.0.000818 PMC9465072 35584003

[24] Bertelli C, et al. IslandViewer 4: expanded prediction of genomic Islands for larger-scale datasets. Nucleic Acids Res. 2017;45(W1):W30–5. 28472413 10.1093/nar/gkx343 PMC5570257

[25] Chakraborty J, et al. Performance assessment of genomic Island prediction tools with an improved version of Design-Island. Comput Biol Chem. 2022;98:107698. 35597186 10.1016/j.compbiolchem.2022.107698

[26] Hudson CM, Lau BY, Williams KP. Islander: a database of precisely mapped genomic Islands in tRNA and TmRNA genes. Nucleic Acids Res. 2015;43(D1):D48–53. 25378302 10.1093/nar/gku1072 PMC4383910

[27] Chan PP, Lowe TM. tRNAscan-SE: searching for tRNA genes in genomic sequences. Methods Mol Biol. 2019;1962:1–14. 31020551 10.1007/978-1-4939-9173-0_1 PMC6768409

[28] Edgar RC. MUSCLE: multiple sequence alignment with high accuracy and high throughput. Nucleic Acids Res. 2004;32(5):1792–7. 15034147 10.1093/nar/gkh340 PMC390337

[29] Galperin MY, et al. Microbial genome analysis: the COG approach. Brief Bioinform. 2019;20(4):1063–70. 28968633 10.1093/bib/bbx117 PMC6781585

[30] McDaniel LD, et al. High frequency of horizontal gene transfer in the oceans. Science. 2010;330(6000):50–50. 20929803 10.1126/science.1192243

[31] da Silva Filho AC, et al. Prediction and analysis in Silico of genomic Islands in Aeromonas hydrophila. Front Microbiol. 2021;12:769380. 34912316 10.3389/fmicb.2021.769380 PMC8667584

[32] Große C, et al. Loss of mobile genomic Islands in Metal-Resistant, Hydrogen-Oxidizing Cupriavidus metallidurans. Appl Environ Microbiol. 2022;88(4):e02048–21. 34910578 10.1128/aem.02048-21 PMC8862790

[33] Maguire F, et al. Metagenome-assembled genome Binning methods with short reads disproportionately fail for plasmids and genomic Islands. Microb Genomics. 2020;6(10):e000436. 10.1099/mgen.0.000436 PMC7660262 33001022

[34] Auvray F, et al. Insights into the acquisition of the Pks Island and production of colibactin in the Escherichia coli population. Microb Genomics. 2021;7(5):000579. 10.1099/mgen.0.000579 PMC8209727 33961542

[35] Giegé R, et al. Structure of transfer rnas: similarity and variability. Wiley Interdisciplinary Reviews: RNA. 2012;3(1):37–61. 21957054 10.1002/wrna.103

[36] Williams KP. Integration sites for genetic elements in prokaryotic tRNA and TmRNA genes: sublocation preference of integrase subfamilies. Nucleic Acids Res. 2002;30(4):866–75. 11842097 10.1093/nar/30.4.866 PMC100330

[37] Lawrence JG, Ochman H. Amelioration of bacterial genomes: rates of change and exchange. J Mol Evol. 1997;44:383–97. 9089078 10.1007/pl00006158

[38] Zhou L, et al. Ggmsa: a visual exploration tool for multiple sequence alignment and associated data. Brief Bioinform. 2022;23(4):bbac222. 35671504 10.1093/bib/bbac222

[39] Mahajan S, Agashe D. Evolutionary jumps in bacterial GC content. G3 Genes|Genomes|Genetics. 2022;12(8):jkac108. 35579351 10.1093/g3journal/jkac108 PMC9339322

[40] Chen H, Skylaris C-K. Analysis of DNA interactions and GC content with energy decomposition in large-scale quantum mechanical calculations. Phys Chem Chem Phys. 2021;23(14):8891–9. 33876048 10.1039/d0cp06630c

[41] Mohapatra B, Malhotra H, Phale PS. *Life Within a Contaminated Niche: Comparative Genomic Analyses of an Integrative Conjugative Element ICEnahCSV86 and Two Genomic Islands From Pseudomonas bharatica CSV86*^*T*^*Suggest Probable Role in Colonization and Adaptation*. Front Microbiol. 2022;13:928848. 35875527 10.3389/fmicb.2022.928848 PMC9298801

[42] Pongchaikul P, et al. AcGI1, a novel genomic Island carrying antibiotic resistance integron In687 in multidrug resistant Achromobacter xylosoxidans in a teaching hospital in Thailand. FEMS Microbiol Lett. 2020;367(14):fnaa109. 32592387 10.1093/femsle/fnaa109 PMC7610039

[43] Ma L, et al. Integrative assessments on molecular taxonomy of Acidiferrobacter thiooxydans ZJ and its environmental adaptation based on mobile genetic elements. Front Microbiol. 2022;13:826829. 35250944 10.3389/fmicb.2022.826829 PMC8889020

[44] Galperin MY, Kolker E. New metrics for comparative genomics. Curr Opin Biotechnol. 2006;17(5):440–7. 16978854 10.1016/j.copbio.2006.08.007 PMC1764326

[45] Morgado SM, Vicente ACP. Exploring tRNA gene cluster in archaea. Memórias Do Instituto Oswaldo Cruz. 2019;114:e180348. 30624459 10.1590/0074-02760180348 PMC6333295

[46] Hu S, et al. Adsorption of Hg2+/Cr6 + by metal-binding proteins heterologously expressed in Escherichia coli. BMC Biotechnol. 2024;24(1):15. 38521922 10.1186/s12896-024-00842-9 PMC10960487

[47] Golyshina OV, et al. ARMAN’ archaea depend on association with euryarchaeal host in culture and in situ. Nat Commun. 2017;8(1):60. 28680072 10.1038/s41467-017-00104-7 PMC5498576

[48] Luo Z-H, et al. Diversity and genomic characterization of a novel parvarchaeota family in acid mine drainage sediments. Front Microbiol. 2020;11:612257. 33408709 10.3389/fmicb.2020.612257 PMC7779479

[49] Srivastava A, et al. Toxin-antitoxin systems and their medical applications: current status and future perspective. Appl Microbiol Biotechnol. 2021;105(5):1803–21. 33582835 10.1007/s00253-021-11134-z

[50] Hampton HG, et al. Functional genomics reveals the toxin–antitoxin repertoire and abie activity in Serratia. Microb Genomics. 2020;6(11):e000458. 10.1099/mgen.0.000458 PMC7725324 33074086

[51] Talwar S, et al. Role of VapBC12 Toxin-Antitoxin locus in Cholesterol-Induced mycobacterial persistence. mSystems. 2020;5(6):10–1128. 10.1128/mSystems.00855-20 PMC7771538 33323416

[52] Peng J, Triplett LR, Sundin GW. Activation of metabolic and stress responses during subtoxic expression of the type I toxin Hok in Erwinia amylovora. BMC Genomics. 2021;22(1):74. 33482720 10.1186/s12864-021-07376-w PMC7821729

[53] Fraikin N, Goormaghtigh F, Van Melderen L. Type II Toxin-Antitoxin systems: evolution and revolutions. J Bacteriol. 2020;202(7):10–1128. 10.1128/JB.00763-19 PMC7167474 31932311

[54] Dai J, et al. MazEF Toxin-Antitoxin System-Mediated DNA damage stress response in Deinococcus radiodurans. Front Genet. 2021;12:632423. 33679894 10.3389/fgene.2021.632423 PMC7933679

[55] Jin C, et al. Structural and functional analysis of the Klebsiella pneumoniae MazEF toxin-antitoxin system. IUCrJ. 2021;8(3):362–71. 10.1107/S2052252521000452 PMC8086154 33953923

[56] Van Gundy T, et al. An antisense RNA Fine-Tunes gene expression of the type II MazEF Toxin-Antitoxin system. mBio. 2022;13(1):e03443–21. 35012340 10.1128/mbio.03443-21 PMC8749433

[57] Dawson CC, et al. Discovery of a novel type IIb RelBE toxin-antitoxin system in Mycobacterium tuberculosis defined by co-regulation with an antisense RNA. Mol Microbiol. 2022;117(6):1419–33. 35526138 10.1111/mmi.14917 PMC9325379

[58] Duperray M, François J-M, Capp J-P. Tuning the expression of the bacterial RelBE toxin–antitoxin system in Saccharomyces cerevisiae allows characterizing the subsequent growth Inhibition. FEMS Yeast Res. 2023;23:foad009. 36722160 10.1093/femsyr/foad009

[59] Chauhan U, Barth C, Valdir, Woychik Nancy A. tRNAfMet inactivating Mycobacterium tuberculosis VapBC Toxin-Antitoxin systems as therapeutic targets. Antimicrob Agents Chemother. 2022;66(5):e01896–21. 35404073 10.1128/aac.01896-21 PMC9116479

[60] Jones SP, et al. Co-expression and purification of a toxin-antitoxin VapC–VapB complex from the E. coli O145:H28 strain RM12581 associated with a Romain lettuce outbreak. FASEB J. 2020;34(S1):1–1.

[61] Zamakhaev M, et al. VapC toxin switches M. smegmatis cells into dormancy through 23S rRNA cleavage. Arch Microbiol. 2022;205(1):28. 36520276 10.1007/s00203-022-03363-1

[62] Chibani CM, et al. Genomic variation among closely related Vibrio alginolyticus strains is located on mobile genetic elements. BMC Genomics. 2020;21(1):354. 32393168 10.1186/s12864-020-6735-5 PMC7216594

[63] Fernández-Gómez B, et al. Patterns and architecture of genomic Islands in marine bacteria. BMC Genomics. 2012;13(1):347. 22839777 10.1186/1471-2164-13-347 PMC3478194

[64] Jia C, et al. Mobilome-driven partitions of the resistome in Salmonella. mSystems. 2023;8(6):e00883–23. 37855620 10.1128/msystems.00883-23 PMC10734508

[65] Kumar A, Das B, Kumar N. Vibrio pathogenicity Island-1: the master determinant of cholera pathogenesis. Front Cell Infect Microbiol. 2020;10:561296. 33123494 10.3389/fcimb.2020.561296 PMC7574455

[66] Seth-Smith H, et al. Chlamydia suis undergoes interclade recombination promoting Tet-island exchange. BMC Genomics. 2024;25(1):724. 39060998 10.1186/s12864-024-10606-6 PMC11282597

[67] Zhang G, et al. The integrase of genomic Island GIsul2 mediates the mobilization of GIsul2 and ISCR-related element CR2-sul2 unit through site-specific recombination. Front Microbiol. 2022;13:905865. 35979485 10.3389/fmicb.2022.905865 PMC9376610

[68] Trzilova D, Tamayo R. Site-Specific Recombination– How simple DNA inversions produce complex phenotypic heterogeneity in bacterial populations. Trends Genet. 2021;37(1):59–72. 33008627 10.1016/j.tig.2020.09.004 PMC7755746

[69] Makarova KS, et al. Evolution and classification of the CRISPR–Cas systems. Nat Rev Microbiol. 2011;9(6):467–77. 21552286 10.1038/nrmicro2577 PMC3380444

