# Supplementary material for: The environmental adaptation of acidophilic archaea: promotion of horizontal gene transfer by genomic islands
Source: BMC Genomics. 2025 Jul 28;26:696. doi: 10.1186/s12864-025-11875-5 (PMC12302457; doi:10.1186/s12864-025-11875-5)
Supplement: Supplementary file 5 — Supplementary Material 5. [file 12864_2025_11875_MOESM5_ESM.docx]

**
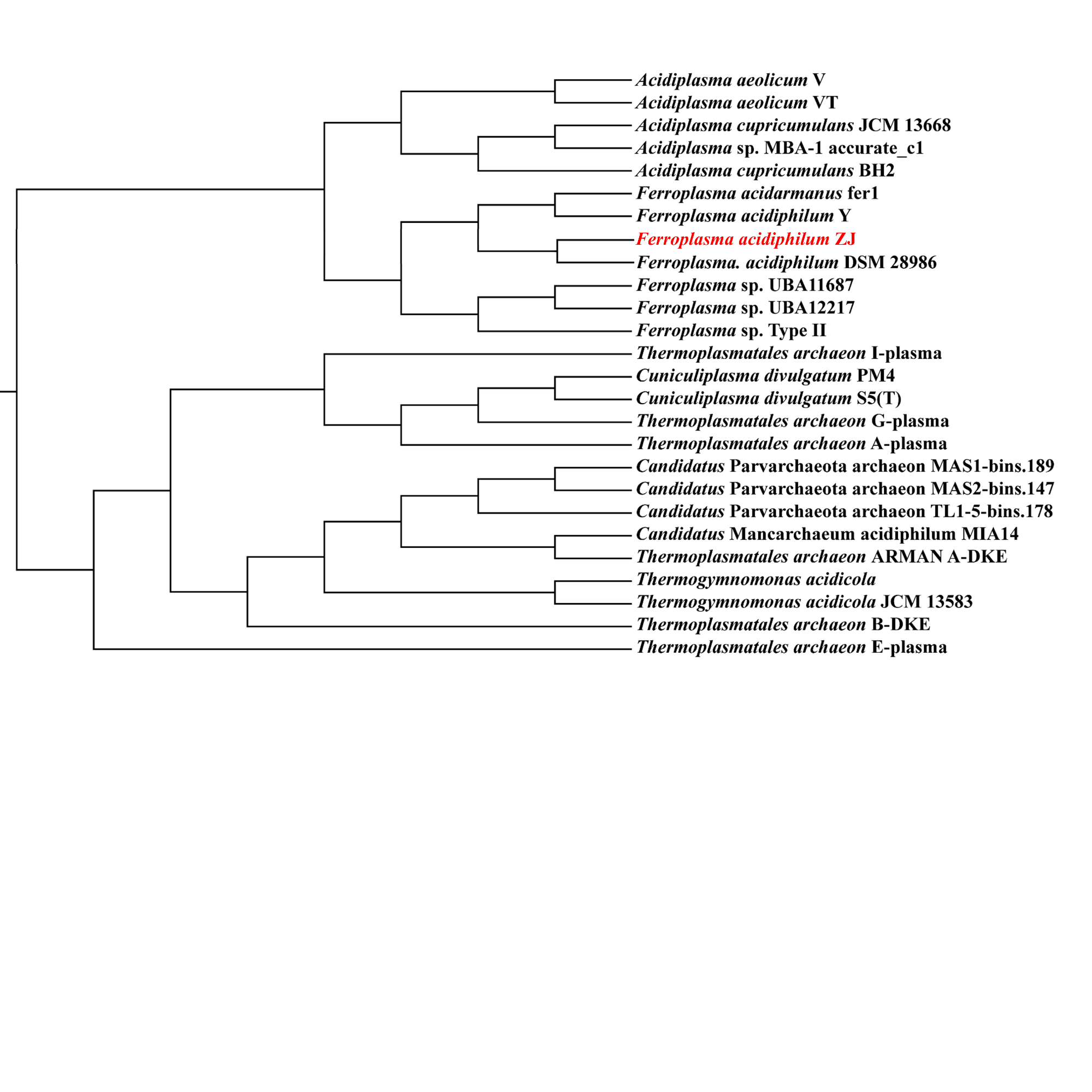
**

**Figure S1**. Phylogenetic tree based on Whole genome of 26 strains.


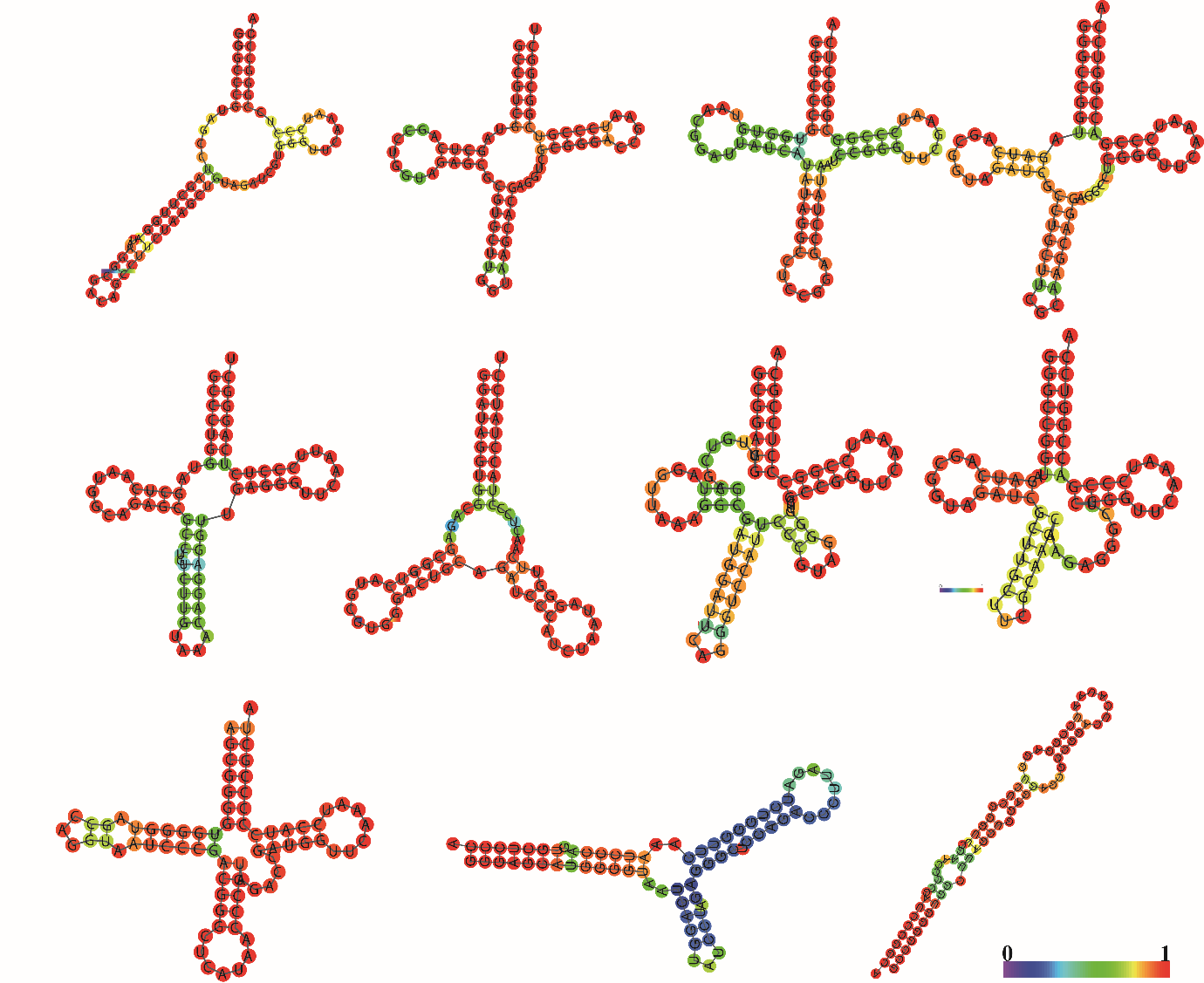
**Figure S2**. Secondary structure of flanked tRNAs.


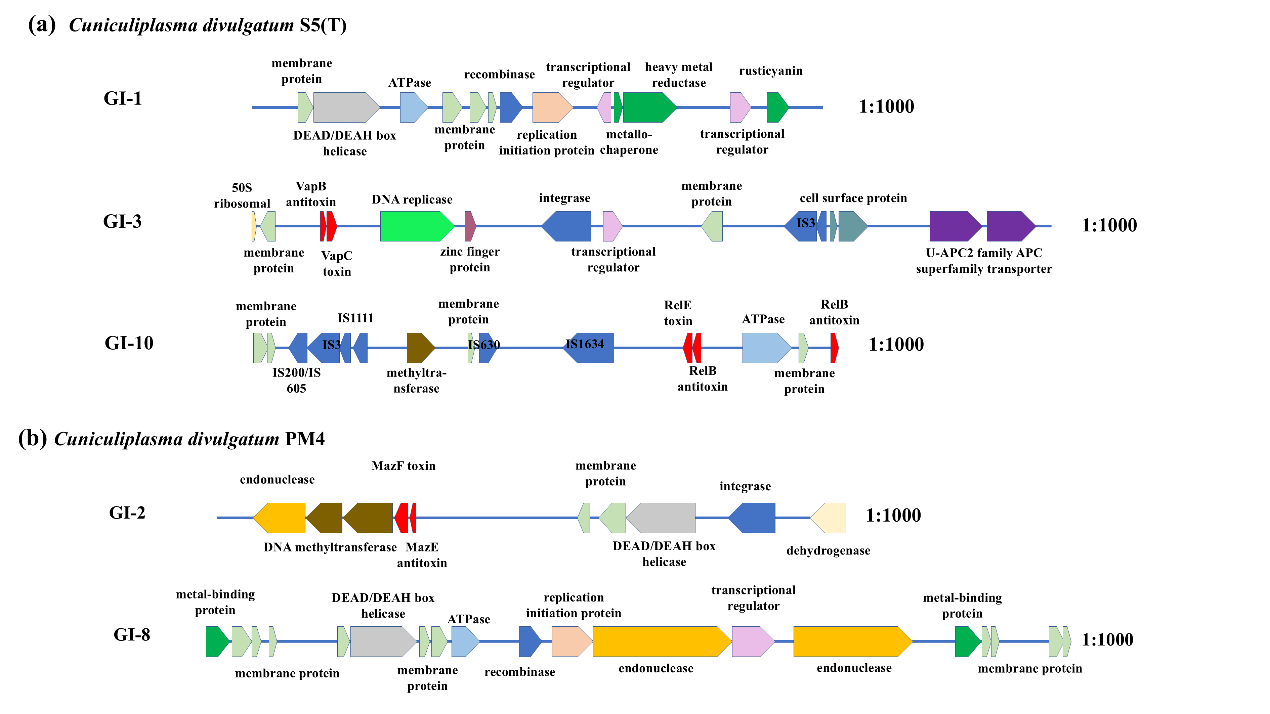


**Figure S3**. Genetic physical map of GIs related to enhance stress resistance
